# Transcranial Magnetic Stimulation-induced Mania: A Risk Worth Taking?

**DOI:** 10.1192/j.eurpsy.2024.1470

**Published:** 2024-08-27

**Authors:** M. Pereira, P. Abreu, M. P. Cameira, M. R. Soares

**Affiliations:** Lisbon Psychiatric Hospital Center, Lisbon, Portugal

## Abstract

**Introduction:**

In the context of treatment-resistant bipolar depression, the use of neuromodulation techniques, notably transcranial magnetic stimulation (TMS), has been on the rise, particularly in the treatment of mood disorders. TMS involves the generation of a strong pulsed magnetic field through an electromagnet placed near the skull, thereby inducing an electrical field capable of depolarizing nerve cell membranes (Dolberg et al., 2001). The magnetic nature of TMS carries advantages compared to direct electric stimulation, such as fewer side effects, reduced invasiveness, and precise targeting. Nevertheless, it is not without its risks. Reported concerns include the induction of manic or hypomanic states, particularly in individuals with bipolar disorder, as well as unipolar depression (Sakkas et al., 2003; Ozten et al.,2013; Knox et al 2021).

**Objectives:**

This review aims to assess the safety and viability of TMS as a therapeutic option and how to best optimize its clinical use.

**Methods:**

A comprehensive literature review was conducted utilizing resources from Pubmed, Researchgate, and Google Scholar.

**Results:**

Despite some inconsistencies and potential confounding factors, our findings suggest that TMS may not significantly elevate the risk of manic switching, especially when compared to conventional treatments like antidepressants. However, it may potentially induce manic episodes, particularly when used as monotherapy or in combination with other treatments. Variables such as treatment protocol and prior response to medication may contribute to mood switching risk. Proposed safety measures include personalized protocol design, close patient monitoring and the combination with mood-stabilizing medication.

**Conclusions:**

Transcranial magnetic stimulation has been associated with manic and hypomanic episodes in mood disorder patients. While the evidence remains limited, it appears that certain individuals may be more susceptible to mood switching. Nevertheless, further research is needed to better elucidate variables influencing mood switching during TMS treatment and to develop effective preventative measures, especially for patients already predisposed to manic switching.

**Disclosure of Interest:**

None Declared

